# Exploring barriers and enablers to implementation of cancer screening among primary care professionals seeing marginalized patients

**DOI:** 10.1186/s12889-025-22835-9

**Published:** 2025-04-28

**Authors:** Arlinda Ruco, Asma Khalil, Cliff Ledwos, Jill Tinmouth, Tara Kiran, Aisha Lofters

**Affiliations:** 1 Interdisciplinary Health Program, St. Francis Xavier University, Antigonish, Nova Scotia, Canada; 2 Peter Gilgan Centre for Women’s Cancers, Women’s College Hospital, Toronto, Ontario Canada; 3 Beatrice Hunter Cancer Research Institute, Halifax, Nova Scotia, Canada; 4 Nova Scotia Health, Halifax, Nova Scotia, Canada; 5 VHA Home HealthCare, Toronto, Ontario Canada; 6 Faculty of Kinesiology and Physical Education, University of Toronto, Toronto, Ontario Canada; 7 Access Alliance Multicultural Health & Community Services, Toronto, Ontario Canada; 8 Sunnybrook Health Sciences Centre, Toronto, Ontario Canada; 9 Institute of Health Policy, Management and Evaluation, University of Toronto, Toronto, Ontario Canada; 10 Department of Family & Community Medicine, University of Toronto, Toronto, Ontario Canada; 11 Department of Family & Community Medicine, St. Michael’s Hospital, Unity Health Toronto, Toronto, Ontario Canada; 12 MAP Centre for Urban Health Solutions, Li Ka Shing Knowledge Institute, St. Michael’s Hospital, Unity Health Toronto, Toronto, Ontario Canada

**Keywords:** Cancer screening, Health equity, Primary care

## Abstract

**Background:**

Cancer screening is an important prevention tool shown to improve cancer morbidity and mortality. Primary care professionals (PCPs) can play an important role in facilitating cancer screening and addressing barriers. Our aim was to learn from PCPs that see a high proportion of patients experiencing marginalization and that have high screening rates in their practices (high performers) to identify key barriers and enablers to addressing cancer screening with this patient population.

**Methods:**

This was a qualitative descriptive study conducted using the principles of ‘design thinking’ to engage PCPs who are high performers in order to understand key barriers and enablers to cancer screening. An interview guide informed by the *Systems Model of Clinical Preventive Care* was used to collect data. Participants eligible for this study included both physicians and nurse practitioners working in Ontario in a variety of settings including solo and team-based practice models. All interviews were audio-recorded, transcribed verbatim and checked for quality assurance. Transcripts were coded by two independent members of the research team using deductive content analysis. The data were mapped onto the *Systems Model of Clinical Preventive Care* domains and presented in a narrative summary.

**Results:**

We interviewed a total of 22 PCPs of which 54.5% were women and just over half (54.5%) were White. Most participants worked in a team-based primary care model. Our results suggest that a number of strategies can support high screening rates among those experiencing marginalization including interprofessional team-based collaborative practice, culturally competent and trauma-informed care, and adaptive approaches to overcome barriers such as improving the ease, access, and acceptability of the screening test.

**Conclusion:**

Addressing cancer screening with patients experiencing marginalization requires a multi-pronged approach to care to facilitate screening. Team-based models of care may have more infrastructure and supports in place to support PCPs in addressing cancer screening with patients experiencing marginalization. Lastly, providers and teams need to work in a supportive clinical context that allows for innovation to address system barriers to promote and enable screening for those who are structurally marginalized.

**Supplementary Information:**

The online version contains supplementary material available at 10.1186/s12889-025-22835-9.

## Background

Cancer screening for breast, cervical and colorectal cancers is an important prevention tool shown to improve cancer morbidity and mortality [1–5]. Organized screening programs have infrastructure in place to invite those eligible for screening, send reminders and track outcomes during the screening trajectory. However, despite the availability of screening programs for breast, cervical and colorectal cancers in many jurisdictions, including the Canadian province of Ontario, a large minority of those who are eligible do not participate in screening [2, 6, 7]. For example, data from 2019 to 2021 show that screening coverage in Ontario was 54.7% for breast cancer, 57.8% for colorectal, and 54.5% for cervical cancer [7]. Moreover, screening disparities have been widely documented for individuals experiencing marginalization such as those with low-income, new immigrants, or those who are part of a sexual or gender minority [8–14]. Recent research also shows that these disparities may have been further exacerbated by the COVID-19 pandemic [15]. 

Primary care professionals (PCPs) can play an important role in facilitating cancer screening and addressing barriers. Studies show that clinician recommendation for screening is one of the strongest and most consistent predictors of screening uptake [16, 17]. PCP continuity may also be important as research shows that patients who rely on walk-in clinics for their primary care have lower cancer screening participation rates [18]. Patients who rely on walk-in clinics are also more likely to be foreign-born and/or living in structurally marginalized neighbourhoods [18]. 

One opportunity that exists to reduce screening disparities is the ability to learn from ‘positive deviants’ [19]. This approach has been widely used in the patient safety and quality improvement field and focuses on identifying individuals/organizations who are high performers and trying to understand what makes them high performing despite the fact that they are also faced with the same constraints as others [19]. Therefore, our aim was to learn from PCPs that see a high proportion of patients experiencing marginalization and that have high screening rates in their practices to identify key barriers and enablers to addressing cancer screening with this patient population. In this context, we defined marginalization broadly and included but not limited it to those with low socioeconomic status, immigrants, those identifying as part of a visible minority or who are racialized, including Indigenous, and/or those who are part of a sexual or gender minority.

## Methods

This was a qualitative descriptive study conducted using the principles of ‘design thinking’ which is an innovative, iterative, user-centred approach to solving problems by understanding users and thus better identifying strategies and solutions from their perspective [20]. Design thinking usually begins with research and empathic engagement of those who are the most knowledgeable about the specific product, service or phenomenon of interest [20]. As such, we conducted semi-structured interviews with PCPs that have high screening rates among their patients who experience marginalization to better understand barriers and enablers to implementing evidence-based approaches to cancer screening within their practice. Ethics approval was received from the Women’s College Hospital Research Ethics Board (#2022-0048-E). Informed consent was also obtained from all participants in the study.

### Study framework

**Fig. 1:**
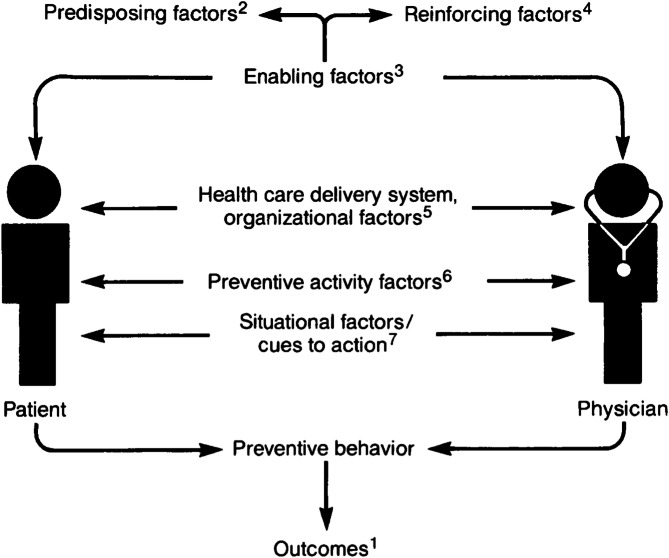
The Systems Model of Clinical Preventive Care by Walsh & McPhee [21]

This study was also informed by the *Systems Model of Clinical Preventive Care* [21] which considers the patient-provider interaction as crucial for health promotion and disease prevention and outlines factors that may impede or enable patients to undertake various preventive care activities such as cancer screening. Specifically, the model outlines how provider and patient predisposing factors (e.g. demographics, health beliefs, ethnicity, history of trauma), reinforcing factors (e.g. social support/approval or social norms, patient satisfaction, case finding), and enabling factors (e.g. education, income, training, technical expertise) can influence preventive behaviour (Fig. 1) [21]. Moreover, the model also recognizes the role of the healthcare system and organizational factors (e.g. access to care, availability of technology/personnel, reimbursement) in taking action. Lastly, the model highlights how situational factors or cues to action (e.g. provider reminders) and factors of the screening test itself (e.g. cost, comfort) can influence one’s decision to engage in that particular preventive activity [21]. 

1– includes preventive care outcomes such as being up to date with cancer screenings.

2– includes factors like demographics, health beliefs, ethnicity, history of trauma.

3– includes factors like education, income, training, technical expertise.

4– includes factors like social support/approval, social norms, patient satisfaction, case finding.

5– includes factors like access to care, availability of technology/personnel, reimbursement.

6– includes factors like cost and comfort.

7– includes factors like patient reminders.

### Participants

Participants eligible for this study were PCPs including both physicians and nurse practitioners working in Ontario in a variety of settings including Community Health Centres (CHCs), Family Health Teams (FHTs), other group practices such as Family Health Organizations (FHOs), or solo practice. CHCs are non-profit organizations that provide interprofessional healthcare services with a focus on primary care, health promotion and community supports for populations who have typically faced barriers accessing healthcare such as those with low income, new immigrants or refugees, and where physicians are paid by salary [22]. FHTs are a team-based model of primary care where a team of non-physician health professionals (nurse practitioners, social workers, psychologists, occupational therapists etc.) collaborates with a group of family physicians to provide primary care services, health promotion and disease prevention largely to patients rostered to that group of family physicians [22, 23]. FHTs receieve money to support these other healthcare professionals and for administration and in turn have more accountability to the Ministry of Health for quality of care provided [22, 23]. All physicians in FHTs belong to a payment model type called Family Health Organizations (FHOs), where they are paid primarily via capitation with some fee-for-service payments and financial incentives for achieving quality standards [22, 23]. Notably, not all group practices that are FHOs are FHTs; these FHO practices would be paid primarily by capitation but would not include the inter-professional team [22, 23]. PCPs working in solo practice are generally reimbursed through a fee-for-service model.

Since we were interested in engaging those who are most knowledgeable about implementing cancer screening strategies with patients experiencing marginalization (‘positive deviants’), we targeted PCPs that self-reported having a high proportion (60%+) of patients experiencing marginalization in some way in their practice and had high cancer screening rates (70%+) for these patients for breast, cervical or colorectal cancer. Previous work looking at cancer screening rates among those with a PCP show average screening rates of 65.8% for breast, 72.2% for cervical and 68.3% for CRC for all patients [24]. Therefore, the research team decided a cut-off of 70% for a definition of “high” was appropriate given the focus on patients experiencing marginalization. We also wanted the clear majority of a provider’s practice to be made up of this patient population and chose a cut-off of 60%. For the purposes of our study, marginalization was defined as those with low socioeconomic status, immigrants, those identifying as part of a visible minority or who are racialized, including Indigenous, and/or those who are part of a sexual (e.g. non-heterosexual) or gender (e.g. transgender) minority as cancer screening disparities have been well documented for these groups [11–14]. 

### Recruitment

We used purposeful sampling based on gender, PCP type and practice setting [25] to recruit PCPs through primary care organizations such as CHCs, FHTs, relevant primary care networks (e.g. Seamless Care Optimizing the Patient Experience which includes a large membership from solo practitioners) and specialized clinics that serve some of the most marginalized patients including those of lower socioeconomic status and recent immigrants [10]. A study recruitment poster was shared with these organizations/contacts, and interested PCPs were asked to email the research team to find out more information about the study. Three reminders were sent out to each organization. Once a PCP contacted the research team, they were provided with an Information Letter about the study and if interested, the research assistant proceeded to confirm eligibility for the study. This included asking interested PCPs if they had high screening rates (70%+) for breast, cervical or colorectal cancer and if their practice includes a high proportion (60%+) of patients experiencing marginalization. Interested and eligible participants were then provided with the Informed Consent Form to review ahead of time and oral informed consent was sought from each PCP prior to the beginning of the scheduled interview.

### Data collection

Data was collected through semi-structured telephone or video interviews. An experienced facilitator used an interview guide (Supplementary File 1) informed by the study framework with open-ended questions and prompts for discussion. The interview guide was first piloted with 2 PCPs to ensure clarity and face validity. The guide focused on providers’ approach to cancer screening and on barriers and enablers to implementing cancer screening with patients experiencing marginalization. We made sure to ask participants about their approaches to cancer screening with each of the types of cancers including breast, cervical, and colorectal cancer since the target populations, screening modalities and processes vary widely for these cancers. We also collected some brief demographic information about each PCP and their practice to summarize the study sample (Supplementary File 2) and for transferability of findings. Participants were provided with a $100 gift card as remuneration for their time. We collected data until saturation.

### Data analysis

Information describing the study participants was summarized using frequency counts and proportions. All interviews were audio-recorded and transcribed to capture the entirety of the interview discussion. Transcripts were also checked for quality assurance by a member of the research team, which included verifying that the transcripts accurately captured what was said during each interview against the audio-recording and removing any identifying information from the file. Transcripts were coded line-by-line by two members of the research team who independently reviewed transcripts and proceeded to code using deductive content analysis [26, 27]. The data were mapped onto the *Systems Model of Clinical Preventive Care* domains. Deductive coding includes developing a pre-defined coding frame that can help focus the coding of transcripts to these pre-identified concepts or categories [26, 27]. Specifically, our codebook (Supplementary File 3) included all of the domains of our study framework and only data that fit our coding frame were coded [26–28]. Therefore, this included looking for predisposing, reinforcing, and enabling factors as well as situational factors/cues to action, organizational factors and preventive activity factors that may enable or hinder a PCP in addressing cancer screening with patients who experience marginalization [21]. Any discrepancies among the two coders were resolved by discussion. The interpretation of the data was also discussed with the broader research team.

### Reflexivity

We conducted this study using the Systems Model of Clinical Preventive Care as the framework to inform our methodology and how we approached this research question. The framework allowed us to focus the study on the key patient, provider and system/organizational factors that can enable or hinder the ability of PCPs to address cancer screening and thus informed our deductive content analysis approach. Using this framework made sense as it captured the complexity of the topic and how our research team conceptualized the concept of cancer screening in primary care. The interviews were completed by a non-clinician who collected the data during her post-doctoral fellowship. As such, this avoided any known power dynamics as interviews were not completed by a potential peer or colleague of the participants which may have hindered participants’ willingness to share more openly. In addition, by virtue of the interviewer not being a clinician themselves, it is possible that this impacted the types of probes that may have been asked during each interview given the limited clinical background of the interviewer. Our research team is comprised of primary care providers, clinician scientists, public health researchers and students, and primary care leaders in a CHC setting. The interprofessional nature of our research team allowed us to explore the interpretation of the data from a variety of perspectives and helped us to map the data to the framework. Our research team also has representation from equity-deserving groups and includes individuals who are racialized, immigrants, and part of a sexual and/or gender minority. Our own experiences and interactions with primary care and the healthcare system overall have also therefore shaped the nuances seen in the data from each of our unique perspectives.

## Results

We interviewed a total of 22 PCPs of which 54.5% were women (Table 1). Most participants (72.7%) were physicians, and the remainder were nurse practitioners. Just over half of our sample (54.5%) was White and 31.8% of participants identified as Asian/South Asian. Most participants worked in a team-based primary care model including 45% who worked in a CHC and 13.6% who worked in a FHT. Participants in our sample reported that on average, 78% of their practice was up to date with their breast and colorectal cancer screenings and 79% of their practice was up to date with their cervical cancer screenings.

**Table 1:** Demographic characteristics of the study sample (*N* = 22)

Variable	Count *N* (%)
Type of PCP	
Physician Nurse Practitioner	16 (72.7) 6 (27.3)
Age	
30–39 40–49 50–59 60–69	9 (40.9) 9 (40.9) 2 (9.1) 2 (9.1)
Gender	
Woman Man Non-binary	12 (54.5) 9 (40.9) 1 (4.5)
Race/ethnic background	
Asian South Asian Middle Eastern White Mixed Prefer Not to Say	4 (18.2) 3 (13.6) 1 (4.5) 12 (54.5) 1 (4.5) 1 (4.5)
Practice Type	
CHC FHT FHO Fee-for-service AFP	10 (45.5) 3 (13.6) 5 (22.7) 3 (13.6) 1 (4.5)

AFP = Alternative Funding Plan

CHC = Community Health Centre

FHO = Family Health Organization

FHT = Family Health Team

PCP = Primary Care Provider

The codes mapped onto the data were the domains of the study framework and included patient and provider predisposing, enabling and reinforing factors, system/organizational factors, preventive activity factors and situational/environmental cues to action. A summary of the findings under each of these codes is presented below.

### Patient predisposing factors

The key themes identified in our data under this domain included prioritization of other health issues, psychosocial barriers and health perceptions, and cultural and health literacy level influences. Participants in the study believed that many patients experiencing marginalization had more immediate concerns or pressing issues that providers felt took precedence over preventive health measures like cancer screening. One healthcare professional (PCP01; solo practice) noted, “there are so many other pressing issues that people are dealing with that sometimes that [screening] falls down on the list.” Additionally, PCPs noted that psychosocial barriers, such as history of trauma, abuse, or mental health issues impacts patients’ willingness or ability to undergo screening. For instance, PCP14 (CHC) described:

“I think of one case which was very challenging at a woman in her 60s, who has schizophrenia and lives in a group home… she really wasn’t. She’s not in a place where she. Can actually do the stool test.”

This illustrates the complex interplay between mental health and screening compliance. Another professional (PCP07; FHT) also noted that for some “patients who’ve had a history of sexual abuse or rape, this exam [pap test] is very challenging.”

Cultural factors and health literacy levels were also found to influence patients’ understanding and acceptance of screening. PCP13 (CHC) noted that “some patients who are new to the country kind of say, well, my previous doctor never did this test so why are we doing it here?” Additionally, the lack of health literacy was observed with some patients not being aware or informed about the need for cancer screening. For example, when speaking about their lesbian patients, PCP02 (CHC) stated, “that specific population will think they don’t need the screening because they’re not having sex with a man, but just making sure they understand that they probably should still screen like everybody else.”

### Patient enabling factors

Accessibility and convenience of screening services, and educational support were identified as key findings under this code. Regarding the physical and logistical accessibility of screening services, PCP01 (solo practice) highlighted the challenge of navigating healthcare facilities, “For mammograms, I think us being able to say, you know, ‘your mammogram is gonna be on the X’th floor,’ and like, you know, not having to send them to another facility [was helpful].” Inaccessible and/or inconvenient locations of screening centers were noted to be particularly important for patients experiencing marginalization. PCP06 (FHO) noted “Just making sure that they’re accessing a center if they need to go somewhere that’s within like, you know, a reasonable commute to them, especially in marginalized populations that maybe don’t have access to a car.” Additionally, one healthcare professional noted:

“Things that require three or more steps - where the patient has to navigate not only my office, but then the breast clinic office or how do I drop off the FIT test to a different place that’s not my primary care center. I think that that often has made it challenging for patients to be able to complete those maneuvers.” (PCP04; FHO).

### Patient reinforcing factors

The importance of trust, relationship-building and patient engagement were identified as key considerations that reinforce screening behaviour. A strong, trusting relationship served to motivate patients to participate in cancer screening and take proactive steps towards preventative care. PCP01 (solo practice) observed,

“I think so much of it comes down to relationships that, you know, building that trust early on in our encounters is really important so that there’s understanding that, you know, we have people’s best interest at heart when we’re making these recommendations.”

Another clinician (PCP07; FHT) noted “For those marginalized patients, I feel like that the fact that they know you and they feel comfortable with you, and you’ve done it before it’s a really important piece.” Additionally, PCP01 (solo practice) highlighted the important role of empowerment in preventive healthcare, “Framing that we provide in some of our initial visits around engaging in preventative care and framing it as a way of helping to support people to stay healthy, so that they can thrive and flourish in their new lives here in Canada.”

### Provider predisposing factors

Healthcare professionals’ motivations and approaches to cancer screening are deeply influenced by their personal experiences and beliefs in the importance of screening. For instance, PCP02 (CHC) shared how personal encounters with cancer, such as a nurse within his practice who had cancer and his own mother’s early diagnosis during COVID, have heightened his commitment to screening, emphasizing, “She [the nurse]’s taking it a lot more seriously now and helping people to get their own screening done” and “I’m so grateful that we found it in her [his mother] early…I’ll tell [my patients] about my own personal experience and maybe that’ll touch on their heartstrings to get checked themselves.” This personal connection to cancer fostered a passionate and persistent approach to advocating for and executing screening practices. Additionally, the belief in the significance of screening propelled healthcare professionals like PCP06 (FHO) and PCP07 (FHT) to proactively incorporate screening discussions and actions into routine patient encounters. PCP06 (FHO) described making cancer screening a part of every visit, “It’s something that I address at essentially, any and every visit…you’re due for a PAP, let’s book you on your way out,” while PCP07 (FHT) took an opportunistic approach, “If someone is in my office [for another reason] and they are overdue for a pap…I’ll just say let’s do it now.”

### Provider enabling factors

Our study results suggest that healthcare professionals’ capacity to screen patients effectively is significantly enhanced by a combination of skills and resources, notably electronic medical records (EMRs), cultural competence and language resources, alongside training in patient-centered and trauma-informed approaches. The utilization of EMRs form the backbone of tracking patient’s screening needs, with PCP07 (FHT) noting “I currently use the Practice Solutions [brand name] EMR and in that EMR, we’ve developed a preventative health screening toolbar.” PCP01 (solo practice) noted “From like an institutional level, having access to interpretation services is needed for all of our care, including cancer screenings,” highlighting the importance of having resources and infrastructure to enable culturally competent care. Professionals in our study also underscored the significance of integrating trauma-informed care. PCP07 (FHT) noted, “when the patient comes or at the first time that I’m doing a pap, I do try to ask them if they have a history of sexual trauma,” underlining the importance of being sensitive to patients’ past experiences. PCP09 (CHC) stated, “so paps for trans men are also an area of focus for me,” and elaborated on the need to “offer them to have a support person with them and also talk to them about the language that they prefer to use,” ensuring that screenings are accessible and respectful. Other simple yet impactful considerations included having physically accessible clinic spaces as noted by PCP10 (FHT), “for some folks who cannot get up on a high table, actually something as simple as having an accessible exam table.”

### Provider reinforcing factors

Professional reinforcing factors, such as monetary incentives and team collaboration, played a crucial role in motivating and supporting engagement in cancer screening. Financial rewards for meeting screening targets were described as a motivator for many PCPs. PCP12 (FHT) noted, “there are these preventive care bonuses… I think at least for an early in my career doctor, the preventive care, financial bonuses are like incentivizing.” PCP04 (FHT) also reflected on past practices where “cancer screening was incentivized to complete, monetarily by the government,” indicating the role of financial rewards in promoting screening activities. Beyond financial incentives, the support and collaboration within healthcare teams was cited as helping to reinforce screening efforts of PCPs. PCP07 (FHT) appreciated the opportunity “to have a discussion around approaches to screening with the other members of the team,” which fostered a collective understanding and approach. PCP13 (CHC) highlighted the advantages of working in a CHC where they “have a lot of support and administrative support…It gets pulled by the team [who is overdue] and then they review it.” PCP16 (CHC) further emphasized the collaborative spirit of a healthcare team in encouraging cancer screening: “everyone’s on board, basically…nurses sometimes catch it [missed screening].”

### Preventive activity factors

Healthcare professionals reported several factors related to the cancer screening tests themselves which acted as barriers. For example, PCP12 (FHT) identified the pain associated with mammograms being a significant deterrent to screening, “I think for mammograms, it’s definitely like the pain that the mammogram procedure causes. That is probably the number one barrier cause. They’ve done it once, they’re like, that was the worst thing ever done and I’m not going to do it again.” Similarly, PCP07 (FHT) and PCP10 (FHT) discussed aversions to Pap tests, “with Pap tests, I mean, obviously it’s a more invasive test and a more intimate test. I have some patients who just don’t like the test because it’s uncomfortable and kind of have refused it”(PCP07; FHT) and “Yeah, something a bit easier and less invasive than a Pap test cuz for some people, that’s what puts them off, right, with the history of trauma, especially postmenopausal women with stenosis.” (PCP10; FHT).

### Situational or environmental cues to action

Situational or environmental cues played a crucial role in prompting both professionals and patients towards action. PCP04 (FHO) shared appreciation for the role of reminders sent out by the provincial cancer agency, Ontario Health (Cancer Care Ontario) to patients due and overdue for screening, “the provincial preventive healthcare reminders have also been helpful.” As described by PCP05 (AFP), “if two years does elapse and the patient hasn’t had their appropriate cancer screening, I gave them permission to send a letter to my patient.” PCP11 (FHO) further confirmed the impact of these reminders on patient engagement, “patients get like reminders from the government, they say they, you know, I got a reminder from the government that I’m due for my pap smear.”

### System or organizational factors

Our findings suggest that the COVID-19 pandemic profoundly disrupted routine healthcare services, significantly impacting cancer screening activities. Healthcare professionals encountered numerous challenges, including delays in receiving test results, staffing shortages, and patient reluctance to visit medical facilities due to fear of infection. One PCP highlighted the extent of disruption, stating, “There was a time when, you know, everything was closed down… clients weren’t able to come in, right? That affected a lot of… cancer screening activities.“(PCP01; solo practice) Another added, “COVID affected so many different levels of healthcare, including staffing, including staffing of the pathologists…We’re seeing like 3–4, five-month delays in getting pap results as a result of COVID” (PCP06; FHO). PCP20 (FHO) also noted, “COVID only really affected the pap tests and also actually mammograms too because patients were often afraid to come in.”

Beyond the immediate impacts of the pandemic, various systemic barriers emerged, including difficulties in reaching specific patient populations, and challenges faced by individuals with mental health conditions. To mitigate these challenges, PCPs implemented a range of adaptive strategies. These included organizational policies for callbacks, collaboration with external labs for uninsured patients, and the deployment of automatic recalls to ensure screenings are conducted in a timely manner. For instance, “We do have organizational policies in place already for callbacks and we have quarterly cancer screening initiatives” (PCP01; solo practice), and “We’ve made an arrangement with DynaCare, which is one of the big lab companies where they do our FIT test for uninsured” (PCP03; CHC). Moreover, a proactive approach to patient care was emphasized, “Every patient is triaged before they are seen by a physician. The triage person is trained that no matter what the patient comes in for, their first thing to do is to ensure that their cancer preventions are up to date” (PCP19; solo practice).

## Discussion

This study sheds light on the role PCPs play in mitigating disparities in cancer screening uptake among patients experiencing marginalization. This qualitative exploration with PCPs in Ontario, Canada identified as ‘positive deviants’ highlights the complexity of factors influencing cancer screening. Our results suggest that a number of strategies can support high screening rates among those experiencing marginalization including interprofessional team-based collaborative practice, culturally competent and trauma-informed care, and adaptive approaches to overcome barriers such as improving the ease, access, and acceptability of the screening test.

### Collaborative approaches to healthcare provision

Many of the participants in our study worked in interprofessional team-based settings and described how a collaborative approach to healthcare provision could support cancer screening uptake. PCPs described how their team-based model of care facilitated a more coordinated and effective practice environment that could dynamically address the diverse needs of patients. Participants in our study also highlighted the importance of a collaborative interprofessional team-based approach as they described the role of interns/practicum students, administrative staff, nurses or other care providers in efforts to identify patients overdue for screening and facilitate the screening process. Those in team-based settings demonstrated a heightened capacity to address barriers to screening as a result of the infrastructure and resources available in these settings. This model leverages diverse expertise and resources to meet patient needs, aligning with the broader recognition of team-based care as a critical component of high-quality primary healthcare.

Our results are in alignment with prior work that explored the effects of interprofessional team-based care on cancer screening rates in Ontario between 2011 and 2022 [29]. Overall, the authors found that, compared with those not practicing in an interprofessional setting, physicians practicing in FHTs had higher rates of breast (+ 2.4%), cervical (+ 2%) and colorectal (+ 0.8%) cancer screening on average per year [29]. Bai et al. [29], also hypothesized that increased screening rates in interprofessional team-based models may be a result of task shifting where other team members may be able to address barriers or facilitate screening for patients. Kiran et al. [30] found that patients in team-based capitation practices had a higher absolute difference in improvement (+ 5.3%) in cervical cancer screening rates between 2001 and 2011 compared to patients in non-team based capitation settings though no effect over time was found for breast or colorectal cancer screening. In their analysis of how team-based care impacts access to healthcare and addresses socioeconomic disparities, Zygmunt et al. [31]. indicate that team-based settings can mitigate access problems and unmet health needs more effectively than solo practices. This is particularly important for practitioners working with marginalized populations. They also note socioeconomic gradients in access problems were significantly reduced in team-based care settings [31], suggesting that this model can enhance equitable access to healthcare services and thus perhaps more equitable access to cancer screenings. Our results are also in alignment with prior work exploring the perspectives of family physicians towards access to lung cancer screening for individuals with low income, which also highlight the value of a team-based approach to care [32]. Though we did not explore strategies to address lung cancer screening in our study, our findings may be transferable to this cancer site.

Research on primary care reform across several jurisdictions suggests that in order for team-based practices to be effective, clear policies on team configuration, leadership, scope of practice, role clarity, and financing are needed [33, 34]. Previous research suggest that team-based care can only reach its full potential when there is alignment between system goals at different levels, combined with strategic investments in resources and compensation models that include all team members [33, 34]. Though outside the scope of this study, it would be interesting to explore the specific structure of team-based practices of PCPs who have high screening rates with patients experiencing marginalization to see the structure of such practices with relation to policies, resources and compensation structures. It would also be interesting to explore whether the ability of PCPs with high screening rates in solo practices stem primarily from individual motivation factors and resourcefulness rather than organizational and infrastructural support from a team-based setting.

### Cultural competence

Our findings underscore the importance of cultural competence and trauma-informed care in facilitating cancer screening. The ability of providers to self-reflect, recognize their biases, and understand the unique experiences of marginalized populations is paramount in order to provide care that is compassionate and sensitive to patients of different cultures. Recent research reinforces the need for culturally competent approaches and highlights the disparities that exist without them even when transitioning to different models of care [8, 32]. Lofters et al. [8] illustrate how the transition from a traditional fee-for-service model to an enhanced fee-for-service model (where some capitation was incorporated) in Ontario was associated with widened screening disparities between immigrants and long-term residents. This suggests that without deliberate, inclusive policies, systemic changes may inadvertently exacerbate existing inequalities even when transitioning to different primary care models. Vahabi et al. [9]. further emphasize this point by demonstrating that mammography screening rates among immigrant women are particularly low, influenced by factors such as physician gender, the physician’s training background, and the lack of enrollment in primary care models. They argue for increased access to culturally competent healthcare providers and systems that understand and address barriers specific to these populations [9]. These findings are in alignment with our results and other work [32] which underscore the importance of culturally competent care as critical for addressing cancer screening with patients experiencing marginalization. Our results also demonstrated the importance of having access to interpretation services, for which only CHCs typically have a formal budget line. Other PCPs in our study also emphasized the importance of safe and inclusive language when providing care for patients that may be a part of a gender minority which constitutes an important consideration for culturally competent care.

### Trauma-Informed care

Our findings suggest that integrating trauma-informed approaches may ensure that care is delivered in a manner that is respectful of and sensitive to the history and experiences of individuals who have faced trauma, thereby enhancing trust and willingness to participate in screening activities. This approach is particularly crucial when dealing with marginalized communities, who often face multiple layers of trauma and barriers to accessing care. While our operational definition of marginalization did not include those with traumatic/violent histories, participants in our study often spoke of patients who in addition to experiencing other types of marginalization also had traumatic/violent histories that informed their experience with screening. The need for trauma-informed care is underscored by the experiences of sexual and gender minority (SGM) patients with cancer which were highlighted by providers in our study. These patients often face unique challenges and disparities in cancer-specific outcomes due to their higher risk of trauma and violence, compounded by historical and ongoing discrimination within the healthcare system [35]. A study by Sinko et al. [35]. highlights the importance of recognizing the widespread impact of trauma, integrating trauma-informed principles into cancer care, and actively resisting re-traumatization to significantly improve the screening, care experiences, and health-related outcomes for SGM patients. Similarly, women experiencing homelessness face disproportionately low cancer screening rates and a higher incidence of cervical cancer. Research by Kohler et al. [36]. reveals that these low screening rates are driven not only by structural barriers but also by high levels of trauma, including sexual violence, which significantly impacts their willingness to undergo screening procedures. They emphasize the importance of trauma-informed care for homeless women, involving understanding their unique experiences, building trust, offering choices and providing a safe and supportive environment for screening [36]. Our findings echo past research which demonstrates the critical role of trauma-informed care in improving cancer screening rates among marginalized populations. For example, providers in our study reiterated the need for a gradual approach to screening, particularly for patients with trauma histories. Some providers mentioned HPV self-sampling as a strategy to reduce re-traumatization. Additionally, PCPs reported that establishing a trusting relationship and addressing other health concerns first often paved the way for successful cancer screening discussions.

### Adaptive strategies to overcome barriers

Lastly, our study reveals the importance of a clinical context that allows for innovation and the implementation of adaptive strategies that may work for particular subgroups of patients. For example, providers in our study described adaptive approaches such as having the provincial screening program mail FIT tests directly to their clinics instead of to a home address to overcome challenges related to homelessness or unstable living situations. Other providers found adaptive ways to cover costs associated with cancer screening or negotiated arrangements with service providers or laboratories to address financial barriers faced by uninsured patients. A clinical practice environment that allows and encourages PCPs to try and implement these adaptive strategies, similar to a quality improvement approach, can enhance accessibility to screening and encourage higher screening participation. Prior work has also alluded to adaptive strategies needed to overcome screening barriers for underserved populations, particularly in the context of the COVID-19 pandemic [37]. The Canadian Partnership Against Cancer also calls for the development of tailored approaches to address cancer screening with underscreened populations such as recent immigrants and those with low income [38]. Specific PCP interventions recommended include the use of reminder and recall systems, education and training on how to facilitate screening with different populations and assessment and feedback tools that can help providers hone their approach to cancer screening with patients experiencing marginalization [38]. It would also be interesting to explore whether PCPs with high screening rates in solo practices are more likely to achieve high screening rates as a result of their resourcefulness and adaptive strategies or as a result of individual motivation factors in comparison to those in team-based settings where the enablers might be organizational support and infrastructure. This information may be helpful for highlighting specific strategies that can be developed for each practice setting.

### Strengths and limitations

The results of this study must be interpreted in light of the study strengths and limitations. We collected data from a variety of PCPs including physicians and nurse practitioners working both in solo as well as interprofessional team-based practice models. Our deductive coding approach allowed us to map our findings to the study framework which focused on the patient, provider, and organizational/system factors that may influence preventive care activities. We also had two coders go through all transcripts and resolved any discrepancies through discussion. We collected data until saturation or when similar experiences were being heard from participants and no new information was emerging from additional participant accounts. We also piloted our interview guide for face validity prior to data collection. However, our study has limitations. Though we recruited PCPs working in a variety of settings, our recruitment efforts were largely centered around those working primarily in an urban setting due to recruitment feasibility. It would be important to explore if these findings are transferable to PCPs working in rural settings. Rural populations face health inequities based on geography, and many Indigenous Canadians reside in rural areas. We also had a limited number of PCPs from solo practice in our sample, which may limit the transferability of these findings. It would be important to explore if enablers to cancer screening differ for PCPs working in solo versus team-based settings. While we were not able to go back to participants to confirm our interpretation of the data, we did discuss the interpretation of the study findings with the broader research team which included representation from primary care. Additionally, we asked participants to self-identify themselves as having high screening rates and seeing a high proportion of patients experiencing marginalization in their practice and we recognize that bias may exist in self-report data and selection bias in those who decided to participate.

## Conclusion

In conclusion, our study results suggest that addressing cancer screening with patients experiencing marginalization requires a multi-pronged approach to care to facilitate screening. Providers need to provide culturally-competent and trauma-informed care and address the ease, accessibility and acceptability of cancer screening in light of other competing priorities. Team-based models of care may have more infrastructure and supports in place to support PCPs in addressing cancer screening with patients experiencing marginalization. Lastly, providers and teams need to work in a supportive clinical context that allows for innovation to address system barriers to promote and enable screening for those who are structurally marginalized. Future work includes utilizing the findings of this study to inform strategies to address cancer screening including through the co-design of potential PCP interventions.

## Electronic supplementary material

Below is the link to the electronic supplementary material.

Supplementary Material 1

Supplementary Material 2

Supplementary Material 3

## Data Availability

The datasets used and/or analysed during the current study are available from the corresponding author on reasonable request.

## Abbreviations

AFP: Alternative Funding Plan
CHC: Community Health Centre
EMR: Electronic Medical Record
FHO: Family Health Organization
FHT: Family Health Team
PCP: Primary Care Professional
SGM: Sexual and Gendery Minority
