# Supplementary material for: Exploring barriers and enablers to implementation of cancer screening among primary care professionals seeing marginalized patients
Source: BMC Public Health. 2025 Apr 28;25:1578. doi: 10.1186/s12889-025-22835-9 (PMC12036152; doi:10.1186/s12889-025-22835-9)
Supplement: Supplementary file 1 — Supplementary Material 1 [file 12889_2025_22835_MOESM1_ESM.docx]

**Supplementary File 1.** Interview guide

Thank you for your interest in participating in this study. Before we begin, I would like to go over the Informed Consent Form and address any questions/concerns you may have.

[Facilitator to review Informed Consent Form with participant]

**Start recording.

[Facilitator to repeat the section on “Documentation of Informed Consent” in its entirety to the participant and request the participant’s verbal consent and record consent on file]

DOCUMENTATION OF INFORMED VERBAL CONSENT

By providing verbal consent, I confirm that:

- This research study has been fully explained to me and all of my questions have been answered to my satisfaction
- I understand the information within this informed consent form
- I do not give up any of my legal rights nor release the investigators or involved institutions from their legal and professional responsibilities
- I agree to take part in Phase 1 of this study

Do you provide verbal consent to participate in this study? [If yes, proceed to interview]

Thank you again for your willingness to participate in this study. The aim for today is to learn more about your approach to cancer screening within your primary care practice. Specifically, we are interested in how you address cancer screening with patients that are experiencing marginalization.

In this context, when we say marginalized patients, we are including those with low socioeconomic status, immigrants, those identifying as part of a visible minority or who are racialized including Indigenous, and/or those who identify as part of a sexual (e.g. lesbian, gay, non-heterosexual) or gender (e.g. transgender, non-binary) minority. Please note that there are no right or wrong answers, we are simply interested in hearing your thoughts and opinions.

Do you have any questions before we begin?

Primary Care Practice

1. Can you tell me a little bit about your practice?

Prompts:

1. What type of setting? (CHC, FHT etc.)
2. Do you work as part of a team or solo practice?
3. Who does your patient population consist of?

Prompts:

1. Do many of your patients live in proximity to where your practice is located?
2. What type of marginalization do your patients experience (low income, Indigenous, new immigrants etc.)?

Cancer Screening Approach Overall

1. Can you tell me a little bit about how you approach cancer screening with your patients?

Prompts:

- 1. How do you confirm whether a patient is up to date with screening?
  2. How do you bring up cancer screening during a patient visit?
  3. How do you communicate screening guidelines/not being up to date?
  4. How do you communicate the expected benefits of screening?
  5. How do you articulate the perceived susceptibility of disease for patients?
  6. Do you use any additional resources/brochures in your approach? If so, which ones?

1. Does your approach to cancer screening differ if you are seeing a patient that is experiencing marginalization? How so? (eg. New immigrant, low income, visible minority etc.)

Prompts:

1. Do you address any patient barriers to screening with marginalized patients that may not be a barrier for non-marginalized patients (e.g. not being able to take time off work, indirect costs of screening etc.)?
2. Would/does your approach change if the patient is home insecure/underhoused? How so?
3. Would/does your approach change for a patient that is racialized/visible minority/Indigenous?
4. Would/does your approach change for a patient that was a new immigrant?
5. Would/does your approach change for a patient that was 2SLGBTQ+?
6. Are there different resources you would use to communicate about cancer screening with these patients? If so, what resources?
7. We are interested in learning about how primary care providers approach screening for breast, cervical and colorectal cancer. Can you describe how your approach to screening with marginalized patients may differ based on the cancer site (i.e. for breast, cervical, vs. colorectal)?

Prompts:

1. How do you address breast cancer screening with marginalized patients?
2. How do you address cervical cancer screening with marginalized patients?
3. How do you address colorectal cancer screening with marginalized patients?

Enablers

1. You have been identified as having high screening rates in your practice with marginalized patients. Can you tell me a little bit about why and how you have been so successful at achieving high screening rates with this patient population? Please think about both personal factors as well as any organizational or broader system factors.

**Facilitator to prompt participant under each of these factors if not mentioned**

Prompts:

1. Predisposing factors – your sociodemographic characteristics, personal health habits, attitudes towards screening
2. Reinforcing factors – finding incident cases, patient satisfaction/experience, performance in comparison to other peers
3. Enabling factors – training/technical expertise, understanding and knowledge of current screening guidelines, logistics like time, staff, space, equipment
4. Health care delivery system/organizational factors – access to primary care, primary care model type, ready availability of preventive services, cost, reimbursement for preventive care, logistics like time, space, equipment, coordination with community resources
5. Test/preventive activity factors – efficacy, efficiency, cost-effectiveness, risks, discomfort, nature of the test (at home vs. not)
6. Situational factors/cues to action – symptoms, provider reminders

Barriers

1. Can you tell me a little bit about any barriers or factors that would prevent you/your colleagues or make it harder for you/your colleagues to address screening with your marginalized patients? Please think about both personal factors as well as organizational or broader system factors.

**Facilitator to prompt participant under each of these factors if not mentioned**

Prompts:

1. Predisposing factors – your sociodemographic characteristics, personal health habits, attitudes towards screening,
2. Reinforcing factors – finding incident cases, patient satisfaction/experience, performance in comparison to other peers
3. Enabling factors – training/technical expertise, understanding and knowledge of current screening guidelines, logistics like time, staff, space, equipment
4. Health care delivery system/organizational factors – access to primary care, primary care model type, ready availability of preventive services, cost, reimbursement for preventive care, logistics like time, space, equipment, coordination with community resources
5. Test/preventive activity factors – efficacy, efficiency, cost-effectiveness, risks, discomfort, nature of the test (at home vs. not)
6. Situational factors/cues to action – symptoms, provider reminders

Other

1. How did the COVID-19 pandemic affect cancer screening in your practice?

Prompts:

- 1. Provincial pauses in screening, switch to virtual care, patients afraid to come in person, competing health care needs

1. Is there anything else that you haven’t already told us about that would be important for other providers to know when addressing cancer screening with marginalized patients?

Thank you once again for your time and insights today.

[Facilitator to stop recording and provide details about the honorarium]
