# Supplementary material for: Exploring barriers and enablers to implementation of cancer screening among primary care professionals seeing marginalized patients
Source: BMC Public Health. 2025 Apr 28;25:1578. doi: 10.1186/s12889-025-22835-9 (PMC12036152; doi:10.1186/s12889-025-22835-9)
Supplement: Supplementary file 2 — Supplementary Material 2 [file 12889_2025_22835_MOESM2_ESM.docx]

**Supplementary File 2.** Participant demographic questionnaire

The following demographic information is being collected for the purposes of describing the group of individuals participating in this study. Any information collected here will only be reported in aggregate form and will not be used to identify any individual persons.

Participant #ID: ___________

1. What is your age?
   - <30
   - 30-39
   - 40-49
   - 50-59
   - 60-69
   - 70+
2. What was your sex assigned at birth?
   - Female
   - Male
   - Intersex
   - Unsure/Do not know
   - Prefer not to answer
3. What is your current gender identity?

- Cis* Woman
- Cis* Man
- Trans** woman
- Trans** man
- Gender fluid
- Gender queer
- Gender nonbinary
- Two-Spirit (Indigenous)
- Another (Specify)__________
- Unsure/Do not know
- Prefer not to answer

*Cis refers to a person whose gender identity is in alignment with the sex they were assigned at birth.

**Trans refers to a person who identifies with a gender other than the one assigned to them at birth, or to a person whose gender identity and gender expression differs from stereotypical masculine and feminine norms. It is also used as an umbrella term for those who identify as transgender, transsexual, trans, gender variant, gender non-conforming, genderqueer, or an analogous term.

1. In our society, people are often described by their race or racial background. These are not based in science, but our race may influence the way we are treated by individuals and institutions. Which category(ies) best describe(s) you? Check all that apply:

- Arab, Middle Eastern or West Asian
- Black (e.g. African, Afro-Caribbean, African Canadian descent)
- East Asian (Chinese, Korean, Japanese, Taiwanese descent)
- Indigenous (First Nations, Metis, Inuk/Inuit)
- Latino/Latina/Latinx
- South Asian (South Asian descent e.g. Indian, Pakistani, Bangladeshi, Sri Lankan, Indo-Caribbean)
- Southeast Asian (Filipino, Vietnamese, Cambodian, Thai, other Southeast Asian descent)
- White (European descent)
- Another category: ________________
- Unsure/Do not know
- Prefer not to answer

1. What are the first 3 digits of the postal code where your primary care practice is located?

_________

1. What is your practice type (CHC, FHT, solo private practice etc.)? ____________________
2. What do you estimate your cancer screening rates to be with your patients?
   - Breast ______ %
   - Cervical ­­­______%
   - Colorectal ­­­______%
