# Supplementary material for: Exploring barriers and enablers to implementation of cancer screening among primary care professionals seeing marginalized patients
Source: BMC Public Health. 2025 Apr 28;25:1578. doi: 10.1186/s12889-025-22835-9 (PMC12036152; doi:10.1186/s12889-025-22835-9)
Supplement: Supplementary file 3 — Supplementary Material 3 [file 12889_2025_22835_MOESM3_ESM.docx]

**Supplementary File 3.** Study codebook derived from the Systems Model of Clinical Preventive Care

| **Code** | **Definition** | **Example(s)** |
| --- | --- | --- |
| Patient predisposing factors | factors which are involved with a patient “wanting to” or being motivated to engage in screening | - demographics (SES, age) - Beliefs and attitudes - Motivation - Expectations - Self-efficacy - Health value orientation |
| Patient enabling factors | skills and resources required by the patient to get screened | - Health Knowledge/education - Skills (literacy) - Logistics (schedule/ convenience) - Income - Physiologic factors |
| Patient reinforcing factors | Support or rewards for engaging in screening | - Social support/ approval - Inherent reinforcement value of screening |
| Provider predisposing factors | Factors which affect a physician’s motivation/desire to engage in screening | - Demographics (ethnicity) - Gender - Language concordance - Personal health habits - Beliefs/Attitudes (self-efficacy, belief that counselling/screening is important, conception about their role) |
| Provider enabling factors | Skills and resources that increase a physician’s likelihood to screen patients | - Training in screening/preventive care (counselling) - Technical expertise - Understanding of goals of screening and current recommendations/ guidelines - Logistics (time, space, staff, equipment) - Availability of materials |
| Provider reinforcing factors | Support or rewards for engaging in screening | - Colleague support and approval - Incentives - Patient satisfaction - Case finding |
| System/organizational factors | Broader system-level or organizational-level barriers or enablers that can promote screening | - Access to medical care - Ready availability of screening (technology/ personnel) - Cost of screening - Organizational priorities - Structure of practice setting - Logistics (time restrictions in the practice, coordination with community resources) - Reimbursements |
| Preventative activity factors | Features of the preventive activity itself | - Costs - Efficacy/effectiveness of test - Efficiency of test - Risks (radiation) - Discomfort |
| Situational/Environmental cues to action | Triggers to the health behaviour (screening) | - Internal cues (symptoms) - External (reminders) |
